# Application of Next-Generation Sequencing to Reveal How Evolutionary Dynamics of Viral Population Shape Dengue Epidemiology

**DOI:** 10.3389/fmicb.2020.01371

**Published:** 2020-06-19

**Authors:** Hui-Ying Ko, Gielenny M. Salem, Gwong-Jen J. Chang, Day-Yu Chao

**Affiliations:** ^1^Graduate Institute of Microbiology and Public Health, College of Veterinary Medicine, National Chung-Hsing University, Taichung, Taiwan; ^2^Arboviral Diseases Branch, Division of Vector-Borne Diseases, Centers for Disease Control and Prevention, Fort Collins, CO, United States

**Keywords:** dengue, evolution, next-generation sequencing, increasing severity epidemiology, genetic variants

## Abstract

Dengue viral (DENV) infection results in a wide spectrum of clinical manifestations from asymptomatic, mild fever to severe hemorrhage diseases upon infection. Severe dengue is the leading cause of pediatric deaths and/or hospitalizations, which are a major public health burden in dengue-endemic or hyperendemic countries. Like other RNA viruses, DENV continues to evolve. Adaptive mutations are obscured by the major consensus sequence (so-called wild-type sequences) and can only be identified once they become the dominant viruses in the virus population, a process that can take months or years. Traditional surveillance systems still rely on Sanger consensus sequencing. However, with the recent advancement of high-throughput next-generation sequencing (NGS) technologies, the genome-wide investigation of virus population within-host and between-hosts becomes achievable. Thus, viral population sequencing by NGS can increase our understanding of the changing epidemiology and evolution of viral genomics at the molecular level. This review focuses on the studies within the recent decade utilizing NGS in different experimental and epidemiological settings to understand how the adaptive evolution of dengue variants shapes the dengue epidemic and disease severity through its transmission. We propose three types of studies that can be pursued in the future to enhance our surveillance for epidemic prediction and better medical management.

## Introduction

Dengue Virus (DENV), a member of the genus *Flavivirus* in the family of *Flaviviridae*, is a single-strand, positive-sense RNA virus (King et al., 2011). Dengue viruses can be classified into four serotypes, DENV-1 to DENV-4. Humans infected with DENV develop only serotype-specific but life-long protective immunity. Antigenic differences between different serotypes limit the cross-protection from other uninfected serotypes. Therefore, secondary or heterologous sequential DENV infections are commonly observed (Guzmán et al., 2002a, b; Yacoub et al., 2013). Humans infected by dengue virus show a wide range of clinical manifestations from asymptomatic, mild fever to severe hemorrhagic diseases. Most DENV infections were asymptomatic or self-limited dengue fever (DF) characterized by fever, joint pain, muscles sore, and severe headache; a small portion of infections manifest the severe form of the disease resulting in dengue hemorrhagic fever (DHF), which is characterized by bleeding, plasma leakage, thrombocytopenia, and increased vascular permeability. Additionally, DHF can lead to fatal complications known as dengue shock syndrome (DSS). The severe dengue is the leading cause of pediatric hospitalizations and death, and a major public health burden in dengue-endemic or hyperendemic countries (World Health Organization [WHO], 2009).

**FIGURE 1:**
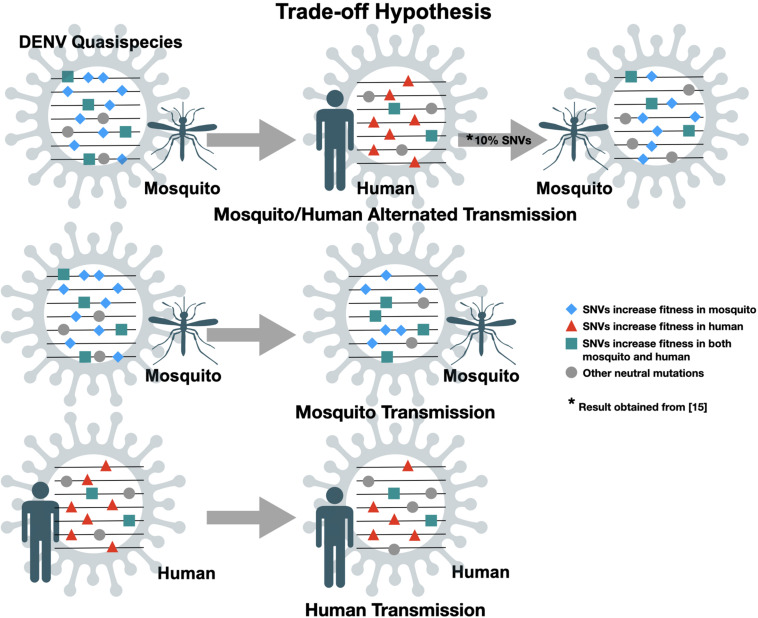
Trade-off hypotheses of dengue virus quasispecies transmission. Random mutations are introduced into virus genome during replication cycles. Transmission involves transferring minor population with variants of genome, practically assayed by single nucleotide variants (SNVs). Only SNVs with increased fitness in both mosquito and human will be kept after mosquito/human alternated transmission.

The genome of DENV contains one single open reading frame (ORF) flanked by 5′ and 3′ untranslated regions (UTR). The ORF is translated as a single, long polypeptide, followed by post-translational process by host and virus-encoded proteases to generate three structural proteins, capsid (C), pre-membrane (prM) and envelope (E), and seven non-structural (NS) proteins, NS1, NS2a, NS2b, NS3, NS4a, NS4b, and NS5 (Chambers et al., 1990). Due to a lack of proofreading function in the RNA-dependent RNA polymerase (RdRp), in each virus replication cycle RdRp will induce random mutations in the genome. Therefore, RNA viruses usually have an exceptionally high mutation rate resulting in the diversification of genomic sequences within an infected individual, so-called intra-host genetic variants or quasispecies. The quasispecies theory, proposed by Manfred Eigen, first illustrates the evolution of self-replicating RNA molecules (Eigen et al., 1989). Detailed implications on virus evolution are reviewed by Domingo and colleagues (Domingo et al., 2012). Like other RNA viruses, DENV continues to evolve and the evolutionary forces acting on the viral population include genetic mechanisms [i.e., mutations and recombination (Holmes et al., 1999)], demographic processes (i.e., genetic drift; Lequime et al., 2016) and natural selection (Bennett et al., 2003; Holmes and Twiddy, 2003). Paradoxically, unlike other single-stranded RNA viruses (such as poliomyelitis virus), DENV undergoes substantially slower rates of evolution. Comparing the non-synonymous variation in mutations between hosts (within the same host) and inter-host (among different hosts) obtained from traditional Sanger consensus sequencing, non-synonymous mutations occurred in viral genome were found more frequently within hosts rather than inter-host implying the strong negative/purified selection of DENVs (Holmes, 2003). The “trade-off” hypothesis (Figure 1) has been postulated to explain this disparity (Vasilakis et al., 2009; Deardorff et al., 2011; Sim et al., 2015; Hanley et al., 2019). Alternating replication between vertebrate and arthropod hosts constrains arbovirus evolution due to the different fitness landscapes shaped by apparently distinct physiological environments within the two different hosts.

## Detecting Genetic Viral Variants by Traditional Sanger Sequencing

The four serotypes of DENVs, which circulated between humans and peridomestic *Aedes* mosquitoes, emerged in four independent events from sylvatic progenitors. The sylvatic strains were maintained in non-human primates and jungle mosquito species within sylvatic cycles (Wang et al., 2000). Although dengue viruses can be transmitted between humans by various jungle mosquito species, such as *Ae. furcifer*, *Ae. Luteocephalus*, and *Ae. taylori*, the urban cycle is maintained by urban mosquito species, mainly by *Ae. aegypti* and *Ae. albopictus* (Chen and Vasilakis, 2011). The urban DENV lineages are ecologically and evolutionarily independent from the ancestral sylvatic viruses, but might undergo selection to increase its virulence in humans. Epidemiological studies showed that globally severe dengue, including DHF and DSS, increased its frequency since its first documentation in the 1950s (Gubler, 2002). Phylogenetic evidence indicates that some DENV strains or genotypes, such as Southeast Asian (SEA) DENV-2 genotype, have the tendency to cause severe diseases. This DENV-2 SEA genotype tends to cause DHF/DSS than the original American genotype after its introduction to the Americas in middle twentieth-century (Rico-Hesse et al., 1997). These traditional studies attempted to address viral genetic variation had to rely on using Sanger sequencing, which was developed by Frederick Sanger in 1977. The method was invented based on the selective incorporation of labeled dideoxynucleotides terminator during DNA replication (Sanger, 1988). Although this has been the most widely performed sequencing method with advantages of convenience, relative longer reads length and lower cost for each reaction, the proportion of genetic variants needs molecular cloning plus Sanger sequencing (Lin et al., 2004). The process is characterized as laborious (Chao et al., 2005; Gong et al., 2013), and biological properties of the genetic variant may directly bias the chance of recovery (Forns et al., 1997). Unfortunately, the current viral surveillance system remains mostly dependent on Sanger sequencing. Adaptive mutations within the minor population are obscured by wild-type sequences and only revealed once they become dominant in the virus population, a process that can take months or years.

## Detecting Genetic Viral Variants by High-Throughput Sequencing

With the advancement of high-throughput next-generation sequencing technologies (NGS), it is achievable to conduct the genome-wide interrogation of virus population within-host (human or mosquito) and transmission between hosts (human and mosquito). High-throughput NGS techniques, including next-generation and third-generation sequencing methods, have been developed with longer reads, greater amount of sequencing data, real-time basecalling with lower cost per reads, aiming to unbiasedly explore the massive information of sequence variations. However, detecting viral variants from NGS data is not straightforward. The process usually involves aligning reads (i.e., short sequence segments) to the reference sequence and compare a spectrum of nucleotides with the reference nucleotide at each position. Between-host variations are determined by constructing the nucleotide sequences with the highest frequency at each position. In contrast, within-host variation usually requires further tests on the distribution of nucleotide at each position. Specifically, statistical methods will be applied to determine if the occurrence of a nucleotide is generated by chance, considering error rate and quality of sequencing. Minor variants are thus defined as nucleotide sequences which are detected with significant frequency but different from the highest frequency at a specific position. Various computational tools have been developed to detect minor variants. Among these tools, LoFreq (Wilm et al., 2012), ViVAN (Isakov et al., 2015), DeepSNV (Gerstung et al., 2012), and Varscan (Koboldt et al., 2009) are widely used in viral genome. Thus far, accurately calling minor variants within viral genome remains a growing field of study.

This review will focus on the NGS studies in different experimental and epidemiological settings to understand how the adaptive evolution of dengue variants shapes the dengue epidemic and disease severity through its transmission. We will discuss the discrepancy of the results between studies and the potential future directions to dissolve the gap.

## Track Genetic Variants Under Experimental Settings

DENV faces different physiological conditions in mosquitoes and humans. Replication cycles between alternate mosquitoes and humans necessitate a fitness trade-off (Vasilakis et al., 2009). To understand how different forces play a role within different hosts requires dissection of the transmission process by either infecting mosquitoes with DENV isolated from humans or infecting humans with DENV isolates from mosquito. Due to ethical considerations, human challenge studies are rarely performed under this purpose (Lyons, 2014; Mammen et al., 2014; Kestelyn et al., 2019); therefore, within-human genetic variations of DENV can only be observed under epidemiological settings. In this section, we reviewed the studies applying NGS to understand the different evolutionary forces acting on mosquito vectors fed with blood meal mixed with dengue virus isolates, or viremic blood directly from dengue patients in the well-defined laboratory settings (Table 1).

**TABLE 1:** Studies utilizing deep sequencing on DENV genetic variants.

	Authors	Epidemic country/year	Virus	Host	Sequencing method	SNV calling method	Intra-host diversity estimation	References
Within mosquito	Lequime S, et al.	Thailand 2010	DENV-1 (*n* = 1 clinical isolate)	*Ae. aegypti*	Illumina full length	LoFreq	Normalized Shannon entropy (S_*n*_) for each nucleotide site Nucleotide diversity at each nucleotide site (π)	Lequime et al., 2016
Mosquito to human	Sim S, et al.	Vietnam 2011	DENV-2 (*n* = 12 patient plasma)	*Ae. aegypti*	Illumina full length	LoFreq	The number of SNVs The sum of SNV frequencies The average SNV frequency The standard error of the mean (SEM) SNV frequency	Sim et al., 2015
	Sessions OM, et al.	Singapore 2005	DENV1 (*n* = 12 clinical isolates)	*Ae. aegypti Ae. albopictus*	Illumina full length	LoFreq	Shannon diversity index Shannon equitability measurements	Sessions et al., 2015
Epidemiology	Parameswaran P, et al.	Nicaragua 2005–2009	DENV-2	Human (*n* = 22) serum	Roche/454 coding region	V-Phaser	A permutation test used to identify specific residues in the genome were hot spots for intra-host diversity. Residues in each genome were scored based their harbored diversity.	Parameswaran et al., 2012
	Ko H-Y, et al.	Taiwan 2001–2003	DENV-2	Human (*n* = 77) serum	Illumina E gene	LoFreq	Number of variants of each sample. Samples divided into two groups by the numbers of variants boundary by medium value.	Ko et al., 2018
	Romano CM, et al.	Brazil 2010	DENV-2	Human (*n* = 11) cultured virus	Roche/454 full length	CLC’s SNP analysis tool	Synonymous changes and non-synonymous changes sits and proportion	Romano et al., 2013
	Rodriguez-Roche R, et al.	Cuba 2001–2002	DENV-3	Human (*n* = 21) cultured virus	Illumina full length	ViVAN	Synonymous variant allele rate Proportion of minor variants > 1%	Rodriguez-Roche et al., 2016
	Parameswaran P, et al.	Nicaragua 2009–2010	DENV-3	Human (*n* = 77) Plasma and PBMC	Illumina full length	In-house python scripts	Percentage of specific loci of nucleotide, codon, and amino acid per protein. Loci indicated each coordinate that was different from consensus sequence.	Parameswaran et al., 2017

### Within Mosquito

**FIGURE 2:**
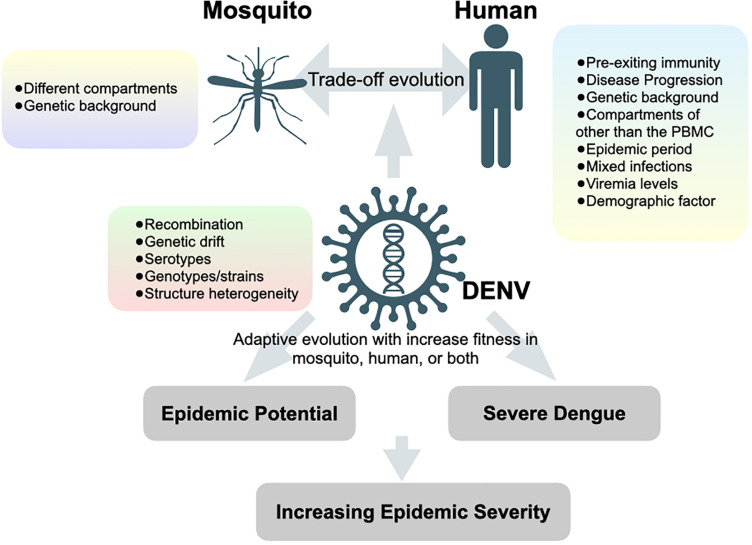
Hypotheses of factors that contributing to increasing epidemic severity. Forces and factors determine the evolution of dengue viruses and the potential outcomes of dengue epidemiology.

Although studies by traditional Sanger sequencing have shown that DENV exists as heterogeneous populations in mosquito vectors (Craig et al., 2003; Lin et al., 2004), the population may differ while virus traverses through different organs of the mosquito (Lequime et al., 2016). Anatomical barriers can lead to population bottlenecks when viruses travel across different organs. Advances in deep sequencing have greatly expanded our ability to examine intra-host diversity by tracking different genetic variants passing through different anatomical barriers of the mosquito vectors. Lequime and colleagues monitored the intra-mosquito evolution of a wild-type dengue virus isolate by infecting mosquitoes with different genetic backgrounds (Lequime et al., 2016). Deep sequencing of full-length viral genomes from various mosquito organs harvested at different time points after infection confirmed that the overall genetic diversity of viral population during infection was predominantly under purifying selection. However, the genetic diversity differed significantly between mosquitoes of different genetic backgrounds despite similar initial bottleneck size. Following a stochastic reduction in genetic diversity, the population size and diversity of DENV rapidly recovered with minor genetic variants after traveling through different compartments and further expanding into the next saliva tissue. In the end, the loss of fitness by inoculating salivary gland-derived virus into the Vero mammalian cell culture confirmed the “trade-off” hypothesis. Since virus diversification could be enhanced by tissue-specific sequence-dependent small interfering RNA (siRNA) during the transversion from the midgut to the saliva of mosquitoes (Brackney et al., 2009), it remains unknown how other factors, such as viral genotypes/strains/serotypes (Parameswaran et al., 2012; Ko et al., 2018) and genetic background in mosquito (Sessions et al., 2015; Grubaugh et al., 2016), would interact with tissue-specific siRNA and impinge on the genetic breadth of virus population as well as its association with the fitness (Figure 2).

### Track Genetic Variant From Human to Mosquito

NGS has been utilized to link the transmission route and to track minor viral variants by single nucleotide variants (SNVs) of intra-host genetic variation of RNA viruses shared by different hosts or individuals (Forbi et al., 2014; Plummer et al., 2015; Nasheri et al., 2017). Sim and colleagues tracked genetic variants transmitted from human to mosquito in the laboratory setting by feeding the mosquitoes, *Aedes aegypti*, with viremic blood from twelve Vietnamese DENV patients (Sim et al., 2015). Viral RNA from both patients’ blood and mosquitoes were analyzed using whole-genome deep sequencing technology. The results showed that the frequency of genetic diversity of DENV, evaluated by different methods based on the SNVs, was similar between human and mosquito. However only approximately one-tenth of SNVs (in a total of 267 SNVs) found in the human was repeatedly shown in the mosquito. This result suggested that most mutations within viremic patients are either deleterious or with poor fitness once transmitted to mosquitoes. Comparing the genetic diversity between two hosts, the genetic mutations of intra-human derived viral variants were mainly located in prM, E and NS1; on the contrary, the genetic mutations of mosquito-derived variants were mainly located in NS3 and 3′ UTR. Similarly, the study by Sessions et al. (2015) using DENV-1 isolated from human viremic sera or two mosquito species after the inoculated intrathoracically found that two residues in the NS5 protein were exclusively acquired from infected *Ae. aegypti* but not from *Ae. albopictus*. The authors also identified a region within the NS3 protein that is refractory to mutations from infected humans and mosquitoes. In summary, although the sequential bottleneck transmission eventually leads to the extinction of many variants, rapid emergence and maintenance of SNVs during DENV transmission from viremic human to mosquitoes were observed, which might lead to the emergence of a new clade or lineage. In the following section, we will review the extent to which the newly evolved clade is associated with disease manifestation during major DENV epidemics.

## Track Genetic Variants Under Epidemiological Settings

Studying intra-host variations under an epidemiological setting aims to provide insight into inter-host evolution through human-mosquito transmission cycles and to give implications of viral fitness, which may lead to increased epidemic potential and/or severe clinical outcomes. Increased epidemic potential and disease severity are primarily influenced by two viral factors: transmissibility and virulence. A deep-sequencing tool was recently applied to study genetic variation and evolutionary dynamics of DENV under different epidemiological settings, which differed in the scale of transmissibility (numbers of infection in the human population) and virulence (numbers of severe dengue cases during the epidemics) (Figure 2). Here, we categorize three major epidemiological phenomena related to viral transmissibility and virulence, commonly observed during DENV outbreaks. We summarize those studies in Table 1 and review below:

### Epidemic Potential

The epidemic potential is the ability of pathogens to rapidly spread and infect many people in the population. Strong purifying selection during alternating transmission between human and mosquito hosts increases the possibility of extinction due to repeated bottleneck transmissions imposed by mosquitoes. Regular lineage extinction and replacement by the variants with better fitness for supporting viral replication in both hosts is one of its dynamic features characterizing the transmissibility of DENV with epidemic potential.

Ko et al. (2018) analyzed viral sequences from the sera of the representative dengue patients during two consecutive DENV-2 outbreaks from 2001 to 2003 in Taiwan. The study identified three consensus genetic variants: groups Ia, Ib, and II, with different spatio-temporal population dynamics. Deep sequencing of the viral envelope gene further confirmed the emergence of those three variants from patient sera. Signature nucleotide changes carried by Ib and II were detected within the quasispecies of Ia virus. The finding indicated the initial Ia variants with low-frequency were gradually replaced by variants of Ib and II with high-frequency or consensus sequence. Combined with the surveillance data and phylogenetic analyses, it showed that no DENV-confirmed cases detected but with group Ib variants continuously circulating during two consecutive years. The observations indicated the potential of group Ib as the “sheltered overwintering” lineage maintained in an undefined ecological niche. On the other hand, group II, which presented higher epidemic potential, was responsible for the larger scale of epidemics during 2002/03. Similar findings were also observed by other group in Singapore studied in between-host DENV-1 diversity during the epidemic in 2013–2014. With more than 40,000 dengue-confirmed cases recorded by the end of 2014, the unprecedented increase in dengue incidence was associated with the switch from DENV-2 to DENV-1 in early 2014. This epidemic strain of DENV-1 initially emerged from an mixed viral population and went through drastically bottleneck transmission with decreased between-host genetic diversity, which led to clade replacement by the emergence of a new lineage of genotype III of DENV-1 (Hapuarachchi et al., 2016). The spatial and temporal dynamics of the inter-human genetic variations of DENV-1 identified by NGS suggests the direct relationship of the numbers of viral variants and the transmission intensity, as reflected by confirmed dengue case-load and transmission hot-spot.

The results from both studies are consistent with the findings by mosquito inoculations as reviewed in section “Track Genetic Variant From Human to Mosquito.” Intensified transmission of DENV may accumulate the viral variants and increase the opportunity of selecting genetic variant(s) with better fitness for replicating both in mosquito and human and lead to the increase of epidemic potential (Romano et al., 2013; Rodriguez-Roche et al., 2016; Ko et al., 2018). The close interaction between viral evolution and transmissibility indicated that tracking genetic diversity during an outbreak is a potential tool to infer DENV transmission dynamics and thereby, to assess the epidemic risk and public health control measures.

### Severe Dengue

The importance of viral genetics in determining the outcome of DENV infection has been suggested in the context of primary versus secondary infections (Vaughn et al., 2000), extent of primary epidemics in naïve populations (Barnes and Rosen, 1974; Gubler et al., 1978), viral variants with different virulence (Rico-Hesse, 2010), sequential order of infecting serotypes (Halstead, 2003), and the influence of pre-existing, heterotypical immunity (OhAinle et al., 2011; OhAinle and Harris, 2014). However, the large body of work has been mainly conducted by the consensus Sanger sequencing. Thus far, five studies have performed to measure genetic diversity within human hosts by deep sequencing and only three studies have considered the epidemiological conditions: clinical features (DF versus DHF) (Parameswaran et al., 2012, 2017) and immune status (primary versus secondary infection) (Rodriguez-Roche et al., 2016; Parameswaran et al., 2017; Ko et al., 2018).

Two studies evaluated the genetic mutation of DENV-3 from patients’ plasma samples and compared the intra-host diversity between DF and DHF cases in Nicaragua within two cohorts (Parameswaran et al., 2012, 2017). The first study on DENV-2 found that the clade replacement of DENV-2, NI-1 clade by a novel NI-2B clade was associated with increased virus replication in native mosquitoes (viral fitness) and clinical severity (Quiner et al., 2014). Further investigation on the patterns of intra-host diversity of the two clades found that direct correlations between viral diversity and clinical outcome were not significant, although NI-2B viruses had lower intra-host diversity than NI-1 viruses (Parameswaran et al., 2012). Consistently, Parameswaran et al. evaluated non-synonymous variants with DENV-3 patient plasma and PBMCs from the cohort during 2009–2010 in Nicaragua, and the results also failed to connect intra-host diversity to disease severity (Parameswaran et al., 2017).

During two separate epidemics caused by DENV-3 and DENV-2 in Cuba and Taiwan, higher genetic variation, defined by arbitrary thresholds of minor variants, was observed in patients with secondary infection than those with primary infection (Rodriguez-Roche et al., 2016; Ko et al., 2018). These studies proposed that the immune status of infected humans is the driving force contributing to the increase in genetic variants and the derivation of antibody-escape mutant(s) as previously suggested by Guzman and colleagues (Guzman et al., 2000), although no experimental data has been provided to verify the results. On the contrary, the study in Nicaragua by Parameswaran et al. found significantly fewer unique loci both in peripheral blood mononuclear cells (PBMC) and plasma obtained from DENV patients with secondary infection than those with primary infection (Parameswaran et al., 2017). It needs to be noted that the strength of positive selection differed in the complete genomic RNAs between PBMC and plasma samples from the same paired DENV patients. Significantly higher abundances of non-synonymous variants were presented in plasma than in PBMCs for prM/M and NS3 regions. Since the prM/M and NS3 regions were suggested to be the major targets of the human B-cell and T-cell immune responses, Parameswaran et al. also concluded that the immune-driven selection pressures could shape the differences in the DENV population released into plasma from PBMC or site(s) supporting viral replication.

In summary, applying NGS technology under epidemiological settings has enhanced our current knowledge on the dynamics of dengue viral population shaped by replication site(s) and serotype-specific immunity, and, in turn, can influence the disease severity during an epidemic. However, the current knowledge gap regarding the association between disease severity and sequence diversity is impeded by different methods and definition in estimating intra-host diversity and the un-controlled confounding factors, such as sampling days, age, immune status with different disease severity, from the epidemiology studies.

#### Increasing Disease Severity Through Epidemics

An increase in the proportion of DHF/DSS cases during the DENV epidemic was first documented in the South Pacific islands in the 1970s (Gubler et al., 1978). Similar epidemiological feature was also reported in several outbreaks in Taiwan (1998, 2002) (Chao et al., 2004; Chen et al., 2008), Cuba (1987, 1997, 2001–2002) (Valdés et al., 1999; Guzmán et al., 2000, 2002b; Peláez et al., 2004; Rodriguez-Roche et al., 2016), Brazil (1981–2002) (Nogueira et al., 2005; Siqueira et al., 2005) and Nicaragua (2004–2008) (OhAinle et al., 2011; Quiner et al., 2014). Deep sequencing technology was applied to explore the dynamic process of viral evolution and its association with increasing clinical severity during the DENV-3 outbreak in Cuba, 2001 (Rodriguez-Roche et al., 2016). This epidemic resulted in 12,889 confirmed cases, including 78 cases of DHF/DSS. A significant monthly increase in the proportion of DHF/DSS cases was observed during this epidemic. A mixed population of DENV-3 within genotype III was identified during the epidemic and the significant number of minor variants were selected and become major variants toward the end of the epidemic. In nostructural protein, the study conducted in Cuba also identified an increasing trend of synonymous variant toward the end of the epidemic, particularly among dengue patients with secondary infections (Rodriguez-Roche et al., 2016). However, the association between specific SNVs or the variability of quasispecies and disease severity was not evident, which was consistent with the other studies (Parameswaran et al., 2012, 2017).

Consistent with the findings of NGS studies from sections “Epidemic Potential” and “Severe Dengue” sections of this review, increasing both the numbers of infected patients and the proportion of DHF/DSS cases usually accompanies each other. This suggests that the gain of fitness by selecting viral variant in favor of replicating in both humans and mosquitoes is accompanied by serial bottleneck transmission during the large DENV outbreak. Prior immunity further contributed to the genetic diversity of DENV due to secondary infection. Selection of variants with growth advantage resulted in a higher probability to cause severe disease during the late phase of the outbreak (Quiner et al., 2014).

## Inferring DENV-Host Interactions From Genetic Variants

Like other viruses, the clinical outcomes of dengue infections were determined by complex interactions between the hosts’ immune status and viral genetics factors (Quinlivan et al., 2007; Tanaka and Mizokami, 2007; OhAinle et al., 2011). Host immunity is expected to exert selective pressure on viral replication. Insect hosts carry several arms of antiviral immunity including RNAi, apoptosis, ubiquitin-proteasome pathway, autophagy, and the heat-shock response (Yadav et al., 2005; Marques and Imler, 2016; Troupin et al., 2016). Rare viral variants having replicative advantages could be selected and become the dominant viral population if their sequence is sufficiently divergent to escape the antiviral immunity (Brackney et al., 2009; Lambrechts and Lequime, 2016).

In addition to innate immunity, antiviral adaptive immunity (in T- and B-cells) in humans can also shape the landscape of viral genomic population. The study in Puerto Rico showed that Clade PR-2B, which emerged during the 1994 DENV-2 epidemic and replaced an endemic PR-1 clade, generated increased levels of subgenomic flavivirus RNA (sfRNA). This sequence-dependent sfRNA is capable of binding to tripartite motif 25 (TRIM25) protein and prevents TRIM25 deubiquitylation, which therefore decreases type I interferon expression through the retinoic acid-inducible gene 1 (RIG-I) pathway (Manokaran et al., 2015).

Intensified transmission of DENV could generate defective interfering (DI) viral particles. Such DI particles have a replication advantage over the full-length parental virus, although they only replicate by complementation in the presence of co-infecting functional viruses. The emergence of defective viruses, including nonsense mutations in the envelope gene, was detected in both humans and mosquitoes over a period of 18 months in Myanmar (Aaskov et al., 2006). Combining dynamic modeling and phylogenetic analysis of viral populations for both intra-host and inter-host dynamics, the results indicated that co-transmission of defective and functional DENV can result in increased transmission and epidemic potential (Ke et al., 2013). These findings provide the underlying mechanism of how the interaction of viral genetic variants and host protein can evade the innate immune response and increases the fitness of the viral population during an epidemic, which leads to an increased epidemic severity.

## Future Perspectives

Efforts on studies of phylogenetic and molecular evolution on viral genomic sequences have provided us valuable insights of DENV evolution and epidemiology within and among different hosts (Bennett et al., 2009; Weaver and Vasilakis, 2009). Although a stochastic process may play a major role in shaping viral genetic diversity, examples of lineage replacement and its association with an increased incidence of DENV infection during outbreaks have been reported (Ko et al., 2018). Furthermore, such genetic variants may also affect the vaccine efficacy, which significantly decreased when the amino acid distance from the DENV-4 vaccine increased and significantly elevated when eight signature positions matched against DENV-4 vaccine strain (Juraska et al., 2018). Unfortunately, such variants with epidemic potential can only be identified retrospectively after the epidemic has occurred due to the wide-scale monitoring of circulating strains is time-consuming and labor-intensive. Here we propose three future directions aiming to establish a link between varying viral diversity and the increased risk of epidemics or severe dengue diseases.

### Using EIP to Predict the Epidemic Potential of Viral Variants

Predicting its evolution toward epidemic potential is complicated by ecological (mosquito) and epidemiological (human) factors. The extrinsic incubation period (EIP), defined as the time interval between pathogen acquisition and pathogen transmission by the vector, is one of the most influential parameters determining the epidemic potential. A study by Fontaine and colleagues found significant variations in EIP in *Aedes aegypti* mosquitoes among eight different isolates of DENV-1, representing the worldwide diversity of recently circulating viruses (Fontaine et al., 2018). Combining *in silico* outbreak simulation, it is predicted that the observed EIP variation in systemic mosquito infection may drive significant differences in the probability of dengue outbreak and the number of human infections. In principle, the shorter the EIP, the higher is the epidemic risk of the virus, potentially resulting in the higher magnitude of dengue outbreaks. If mosquito infection study is not possible for the lab, examining the phylogenetic tree to check any new clade formation may also give a hint of the emergence of potential epidemic strain.

### Establish a Mosquito-Mouse Transmission Model

Another attempt to follow evolutionary trajectories during virus transmission between humans and mosquito recapitulating the emergence of genetic variants with epidemic potential can be found for the Chikungunya virus (CHIKV). CHIKV, normally circulating in *Aedes aegypti* mosquitoes, occurred in the Indian Ocean islands before the 2005–2006 epidemic. It was found that an alanine-to-valine mutation at residue 226 of the E1 glycoprotein (A226V) enhanced the infectivity of another mosquito vector, *Aedes albopictus*, and expanded the affected geographical area (Tsetsarkin et al., 2007). Stapleford and colleagues studied the spatial and temporal evolution of CHIKV during natural transmission between mosquitoes and mice. Their experiments recapitulated the emergence of V80I: A129V from currently circulating A226V variants in the saliva of multiple mosquito strains (Stapleford et al., 2014). More adaptive mutations can also be found while further alternating the virus transmission between two hosts. This mosquito-mouse transmission model could be a useful tool to predict evolutionary trajectories and epidemic potentials of viral variants.

### Big Data Analysis Through Shared NGS and Epidemiological Database

Replication of DENV within human host generates genetic variants of the viral population (intra-host diversity), which are thought to serve as a template on which evolutionary mechanisms, such as recombination, genetic drift, bottlenecking, or positive/negative selective pressure, acts to shape variation at the consensus level between hosts (inter-host diversity). The disease outcomes of viral evolutionary dynamics are determined by multiple factors interplayed between host and virus. However, direct comparisons between different epidemiological studies are difficult, mainly due to the following reasons: (1) the confounding factors from each epidemiological study, (2) different SNV basecalling method used, and (3) intra-host diversity estimation analyzed (Table 1). First, the studies in Cuba and Taiwan had more adult dengue cases, which were different from the study conducted in Nicaragua with mostly pediatric cases. A previous study has shown that asymptomatic or pre-symptomatic patients retain transmissibility to the mosquito, and at a given level of viremia, asymptomatic and pre-symptomatic people significantly and more readily transmit the virus to mosquitoes than symptomatic individuals (Duong et al., 2015). Given the possible high asymptomatic rate of dengue infections (Shepard et al., 2004), the viral intra-host variation within asymptomatic or pre-symptomatic humans is not only a missing part of studying viral micro-evolution but also an important question regarding virus pathogenesis. Second, the SNV calling methods and the statistical methods to infer the frequency of intra-host diversity might also drastically influence the results of epidemiological or clinical inferences. Despite variants calling pipelines such as LoFreq and DeepSNV have been commonly used, the accuracy in characterizing intra-host diversity has rarely been validated under the same sample condition (Barbezange et al., 2018) and it might be sensitive to the input virus titers and the setup of suitable quality thresholds (McCrone and Lauring, 2016). Third, studies in Cuba and Taiwan considered SNV in nucleotide level using the SNV counts or proportions to estimate intra-host diversity, and the study in Nicaragua considered the amino acid level by defining unique loci with non-synonymous minor variants to estimate the intra-host diversity. Currently, there’s no common rules or best approaches to estimate intra-host diversity, which make direct comparison between different epidemiological studies difficult and warrants further investigation.

Recently, more and more studies have applied deep sequencing technique to obtain consensus sequences in individual level. However, rich information embeded in output data have not been fully explored. Data deposited in Sequence Read Archive (SRA) under NCBI, for example, could therefore become a useful resource for researchers to adress clinical or epidmiological questions. Applying deep learning technology on big data analysis will be crucial to elucidate underlying mechanisms by integrating intra-host sequencing data with a systemic collection of epidemiological data acquired from DENV-infected individuals with or without symptoms.

## Author Contributions

H-YK and GS reviewed and drafted the manuscript. G-JC commented and edited the manuscript. D-YC coordinated the review, edited and wrote the manuscript. All authors contributed to the article and approved the submitted version.

## Conflict of Interest

The authors declare that the research was conducted in the absence of any commercial or financial relationships that could be construed as a potential conflict of interest.
